# From Efficacy to Effectiveness and Beyond: What Next for Brief Interventions in Primary Care?

**DOI:** 10.3389/fpsyt.2014.00113

**Published:** 2014-08-28

**Authors:** Amy O’Donnell, Paul Wallace, Eileen Kaner

**Affiliations:** ^1^Institute of Health and Society, Newcastle University, Newcastle upon Tyne, UK; ^2^Department of Primary Care and Population Health, University College London, London, UK

**Keywords:** brief alcohol intervention, efficacy, effectiveness, implementation, research needs, secondary prevention, primary care

## Abstract

**Background:** Robust evidence supports the effectiveness of screening and brief alcohol interventions in primary healthcare. However, lack of understanding about their “active ingredients” and concerns over the extent to which current approaches remain faithful to their original theoretical roots has led some to demand a cautious approach to future roll-out pending further research. Against this background, this paper provides a timely overview of the development of the brief alcohol intervention evidence base to assess the extent to which it has achieved the four key levels of intervention research: efficacy, effectiveness, implementation, and demonstration.

**Methods:** Narrative overview based on (1) the results of a review of systematic reviews and meta-analyses of the effectiveness of brief alcohol intervention in primary healthcare and (2) synthesis of the findings of key additional primary studies on the improvement and evaluation of brief alcohol intervention implementation in routine primary healthcare.

**Results:** The brief intervention field seems to constitute an almost perfect example of the evaluation of a complex intervention. Early evaluations of screening and brief intervention approaches included more tightly controlled efficacy trials and have been followed by more pragmatic trials of effectiveness in routine clinical practice. Most recently, attention has shifted to dissemination, implementation, and wider-scale roll-out. However, delivery in routine primary health remains inconsistent, with an identified knowledge gap around how to successfully embed brief alcohol intervention approaches in mainstream care, and as yet unanswered questions concerning what specific intervention component prompt the positive changes in alcohol consumption.

**Conclusion:** Both the efficacy and effectiveness of brief alcohol interventions have been comprehensively demonstrated, and intervention effects seem replicable and stable over time, and across different study contexts. Thus, while unanswered questions remain, given the positive evidence amassed to date, research efforts should maintain a continued focus on promoting sustained implementation of screening and brief alcohol intervention approaches in primary care to ensure that those who might benefit from screening and brief alcohol interventions actually receive such support.

## Introduction

Brief interventions for alcohol provide a clinically effective and cost-effective means of identifying and addressing alcohol-related problems when delivered in primary healthcare settings (1–4). Originating in the field of smoking cessation (5), and grounded in social cognitive theory (6), brief alcohol interventions aim to detect problems at an early stage, when they are most amenable to adjustment, to promote positive behavior change (7), and thus avoid the development of more serious future problems in an individual (8).

### What is the basis for brief interventions for alcohol?

Brief intervention comprises two broad modalities. First, simple structured advice in the form of personalized feedback on how to address problematic drinking behavior as well as information and/or advice on how to avoid its adverse consequences. This form of intervention is typically delivered in one to five sessions, which are short in duration [a review by Kaner et al. who found a mean of 25 min per intervention (9)]. Second, extended or more intensive intervention, using counselling and other psycho-therapeutic techniques such as motivational interviewing or cognitive behavioural therapy (CBT), which may extend up to 50 minutes per session (9, 10). These more intensive interventions may be delivered either in a single appointment, or via a series of related sessions and the overall treatment exposure has been reported to be 60–175 minutes overall (9). Nevertheless, while the content and delivery style of brief interventions may vary, all are designed to promote awareness of the negative effects of drinking and to motivate positive behavior change (11). The core elements of brief alcohol intervention are based on “FRAMES” (*F*eedback, *R*esponsibility, *A*dvice, *M*enu, *E*mpathy, and *S*elf-efficacy) principles (12), and important components include drawing out individuals’ beliefs and attitudes about drinking, their self-efficacy or sense of personal confidence about changing their drinking, and a view about how their drinking sits in relation to other people’s drinking behavior (normative comparison) (13).

### What are the overall findings from the evidence?

From the first study of the effects of opportunistic brief intervention carried out in Malmo, Sweden in the early 1980s (14) over three decades of research has been undertaken both locally and internationally to develop these simple technologies to assist with the identification of individuals at risk from their alcohol consumption, and the delivery of short, cost-effective interventions in community and health-care settings. A recent review of systematic reviews, covering a total of 56 unique primary healthcare-based randomized controlled trials, found consistent evidence for the effectiveness of brief alcohol interventions in reducing hazardous and harmful drinking when delivered in primary care settings (15). However, some of the more recent individual large scale pragmatic trials have failed to demonstrate significant differences between the effect sizes in the control and intervention groups (16, 17). In addition, as Heather has emphasized, while the evidence base for the implementation of brief structured advice as a form of opportunistic ASBI appears reasonably sound, this is not the case for extended, intensive interventions based more explicitly on motivational interviewing principles (18), despite the sound theory informing such approaches (19).

### Why might some studies fail to find significant treatment effects?

A consistent trend in trials of brief intervention is that of reduction of alcohol consumption in both the control and active intervention conditions (20–22). It is not yet clear if this is due to an artifact of participating in the research process itself (see below) or a response to active ingredients of behavior change, which may be provided to participants allocated to the control condition (23). These include feedback, provision of bibliometric information, and cursory advice about alcohol, which may be embedded with other lifestyle behaviors such as smoking or physical activity. Despite awareness of these issues, there has been little progress to address concerns about assessment reactivity, for example, through the use of Solomon 4-group designs (24–26).

### What do we know about the key elements of intervention and how well they are delivered?

Whilst there is undoubtedly a considerable and largely convincing body of literature supporting the overall effectiveness of brief interventions for alcohol, as the recent McCambridge review confirmed, our understanding of their “active ingredients” remains limited (27). Evidence suggests that for interventions to achieve statistically significant improvements in alcohol outcomes, they should include at least two of the following three elements – feedback, advice, and goal-setting (28). However, the results of a study by Bertholet and colleagues were far less clear-cut, finding that across different populations and settings, intervention characteristics viewed as central to efficacious brief motivational interviews were inconsistent predictors of drinking outcome (29). Further, as both Whitlock and Beich have emphasized (28, 30), given the inevitable “helping relationship” that exists between patient and practitioner, it remains challenging to isolate the impact that the additional support general practitioners might have on intervention effectiveness, particularly when such interventions may be delivered on multiple occasions, and via multiple modalities. There has been some recent work that focused on specific behavior change techniques embedded in advice or counseling (31) but we are not closer to understanding potential therapist effects (either skill, rapport-building, or trust) or the interaction between intervention *per se* and other aspects of recipients’ lives (policy, corporate behavior, and family or personal context). There are also concerns as to whether current brief intervention approaches remain faithful to their theoretical roots. It has therefore been suggested that poor delivery of brief interventions coupled with potential content drift, should result in a cautious approach to future roll-out, whilst additional research is carried out to establish the efficacy of individual intervention components more conclusively (29).

Taking all the above considerations into account, it seems timely to review the current state of the screening and brief alcohol intervention evidence base to determine the extent to which further research is actually required, and to consider which research questions such studies might most usefully examine. After all, any additional research must build upon previous work to save public time and money. For “while replication is an important part of the scientific method, a field needs to progress rather than merely generate volume” (32). Importantly, the development, evaluation, initial adoption, and wider roll-out of a new health intervention or treatment should ideally be supported by a sequence of research studies, ranging from basic “proof of concept” research to demonstration studies. Flay identifies four key levels of experimental research: efficacy (or explanatory) trials; treatment effectiveness (or pragmatic) studies; implementation studies; and finally, program evaluation (or demonstration) research to measure the actual impact of an intervention at wider population level once an intervention becomes part of large scale, mainstream care (33, 34). These levels are both interlinked and interdependent, thus most research is best conceptualized as existing on a continuum, from optimized to naturalistic conditions, as opposed to being easily positioned within one distinct study category (35). Crucially, however, efficacy must be demonstrated before effectiveness is assessed, and the latter is a necessary pre-condition for wider dissemination and subsequent adoption (36).

Against this background, this paper aims to provide an overview of the development of the screening and brief alcohol intervention research field in primary health care drawing primarily on published systematic reviews in the field, supplemented with key recent literature to ensure the evidence presented reflects the cutting edge of this field. In doing so, it will assess the extent to which the existing ASBI evidence base has achieved Flay’s four key levels of intervention research (efficacy → effectiveness → implementation → demonstration) (33, 34), which in turn, will help highlight any outstanding questions for future research.

## Methods

First, the paper draws on the results of a recent overview of systematic reviews and meta-analyses of the effectiveness of brief alcohol intervention in this setting (15). This overview searched key electronic databases (MEDLINE, EMBASE, PsycInfo, The Cochrane Database, The Database of Abstracts of Reviews of Reviews, and the Alcohol and Alcohol Problems Science Database) for systematic reviews and meta-analyses of studies examining the effectiveness of brief alcohol intervention in comparison to control conditions in primary healthcare settings, which were published between 2002 and 2012. Second, the paper synthesizes the findings of more recently published primary studies focused on the improvement and evaluation of the implementation of brief interventions for alcohol in routine primary healthcare to ensure the presented evidence reflects the state-of-the-art in this field.

For the purposes of this paper, primary healthcare has been operationalized to include all immediately accessible general healthcare facilities but not emergency settings. Brief intervention comprises a single session and/or up to a maximum of five sessions of engagement with a patient, and the provision of information and advice designed to achieve a reduction in risky alcohol consumption or alcohol-related problems. Heavy drinking is defined as drinking in excess of 60 g of alcohol per day for men and 40 g for women (37). Hazardous drinking is consumption at a level, or in such a pattern, that increases an individual’s risk of physical or psychological consequences (38), while harmful drinking is defined by the presence of these consequences (39). Alcohol consumption, at a dependent level, results in repetitive problems, affecting three or more areas of life, including a strong desire or compulsion to use alcohol, inability to control use, and withdrawal from and tolerance to alcohol (40).

## Results

### Efficacy, effectiveness, implementation, and programe evaluation: the four phases of brief alcohol intervention research

#### Level 1: Do brief interventions work? Efficacy studies on brief alcohol interventions

An efficacy trial is designed to evaluate what an intervention achieves under optimum conditions (33). It provides a test of (a) a well-specified and standardized treatment or therapy that (b) is made available in a uniform fashion, within standardized contexts or settings, to a specific target audience, which (c) completely accepts, participates in, complies with, or adheres to the treatment/programe as delivered (33). According to the US Society for Prevention Research (36, 41), efficacy testing necessitates the conduct of a minimum of two robust trials [defined as those which include tightly defined populations; psychometrically reliable measures and data collection procedures; rigorous statistical analysis; consistent positive effects (without adverse impacts); and one or more long-term follow-ups]. The randomized controlled trial is generally considered to be the “gold-standard” for intervention evaluation in medical research and the most rigorous way of determining whether a cause–effect relation exists between treatment and outcome (42). This is because this methodological approach is specifically designed to minimize bias and potentially confounding variables through randomization of study participants to prevent systematic differences between intervention groups in any factors (both known and unknown); and double blinding to ensure that the preconceived views of subjects and/or clinicians cannot systematically bias the assessment of outcomes (43).

Clinical drug trials, where a discrete dose of a pharmacological agent is delivered to patients, face fewer challenges in meeting the required standards of treatment efficacy. For behavioral interventions, which generally involve significant inter-personal interaction in the delivery and receipt of advice or counseling, the conditions are more challenging. There is inherent complexity where human actors are required to be a substantial part of “the therapy” (44). Although it can be argued that practitioners often deliver and/or explain the pharmaco-therapy in drug trials, the tablet or pill is generally regarded as the key active ingredient not the explanation or advice *per se*. Despite this challenge, an attempt has been made to disaggregate the component parts of brief alcohol intervention in trial-based evaluations (by characteristics of practitioners, patients, delivery settings, intervention content, scope for flexibility, skill-based training, implementation support, and fidelity monitoring) to assess the extent to which trial-based evaluations show features of uniformity and standardization (efficacy) or not (9). The conclusion of this work was that evaluations in this field sit on a continuum from efficacy to effectiveness trials, because a perfect model of either extreme is hard to achieve. In general, the older trials, which tended to include more tightly controlled evaluations with high levels of internal validity, demonstrated consistently positive outcome effects. Moreover, a series of sensitivity analyses excluding trials with less than adequate features of methodology found persistently positive outcomes. Thus, proof of concept via efficacy trials seems to have been comprehensively demonstrated (45) and more recently re-confirmed by a further systematic review by Jonas et al. (46).

#### Level 2: Do brief interventions work in the real world of primary care? Effectiveness trials

Efficacy trials can establish whether an intervention works (or does more good than harm) when delivered in optimum conditions; effectiveness trials determine whether those benefits continue to be realized in more real-world settings. Sufficient replicability and stability of effects are important aspects of this work especially in “typical” conditions of delivery where availability, compliance or acceptance, and measurement factor may vary (33). As Flay writes *“*an intervention will be effective only if an efficacious treatment/program is delivered/implemented in such a way as to be made available to an appropriate target audients in a manner acceptable to them (i.e. that they will be receptive to, participate in, comply with, or adhere to)” (33).

A recent review of reviews identified at least 56 separate randomized controlled trials of screening and brief alcohol interventions in primary health care, which consistently reported that brief alcohol interventions are effective at reducing hazardous and harmful drinking in primary healthcare, with weekly alcohol consumption the most commonly reported outcome (15). A key issue here, is the size of the outcome effect and the extent to which it is diminished (or not) in more variable pragmatic evaluations. In 2007, meta-analysis of the results from 25 RCTs of screening and brief intervention by Kaner et al. (9) reported an average reduction in the quantity of alcohol drunk of 38 g/week for brief intervention compared with control conditions [95% CI (confidence interval): 23–54 g]. More recently, analysis of the pooled results from 23 RCTs and 6 systematic reviews by Jonas et al. (46) found a slightly increased reduction of 49 g/week for adults aged 18–64 (95% CI: 33–66 g). Thus outcome effects appear to have been generally stable over time as trials have become increasingly pragmatic in nature (9). Finally, in addition to reduced alcohol consumption, this field of work has regularly reported reductions in other outcomes such as alcohol-related problems (9) and reduced health-care utilization (47) and mortality (48). Importantly, delivery by a range of practitioners in primary healthcare settings has beneficial effects (49), although findings of one review suggest that the effect sizes are greater if delivered by doctors (50). In summary, there appears to be ample evidence of replicability and consistency of effects on a number of parameters.

This said, while the overall evidence base seems to show that brief alcohol interventions are both efficacious and effective when delivered in primary care settings, some individual large scale pragmatic trials have reported null findings. For example, a recent large UK trial (SIPS) reported no significant differences in hazardous and harmful drinking status in patients receiving simple feedback after screening plus a patient information leaflet (the control condition), those receiving 5 min of structured advice, and those receiving a further 20 min brief lifestyle counseling (16). This finding accords with three systematic reviews that focused on control conditions only and found consistently reduced drinking in these groups over time (20–22). It may be that the mere fact of participation in a brief intervention trial may be associated with positive behavior change. This may be due to a general “Hawthorn effect,” whereby increased attention or scrutiny might influence drinking (51). It may be that most individuals who agree to participate in a trial have already started a change process. Moreover, given the fact that extreme measures of behavior tend to shift to less extreme positions over time (known as regression to the mean), such reductions in control groups may also be explained by natural reductions in heavy drinking over time (52). Finally, there is growing evidence to suggest that patients’ reactions to the screening or measurement activity itself could influence their decision to cut down their alcohol consumption (known as assessment reactivity) (53, 54). Conversely, while it is possible that individuals with lower reported levels of consumption might increase their drinking over time, this is rarely captured in alcohol trials where only risky drinkers are included at enrolment. Nevertheless, an interesting trend in this field is that the definition of heavier or risky drinkers seems to have been falling over time (9). For example in a 2007 review, average weekly consumption at enrollment (baseline) was 55 standard drink units in the earliest trial (55) but was only 25 standard drink units in the most recent trial (16). Hence, it is possible that the scope for regression to the mean might be reducing in this field. Furthermore, the cumulative (pooled) meta-analyses reported in successive systematic reviews reveal positive outcome effects “over and above” those seen or expected in control conditions (15).

#### Level 3: What factors promote widespread adoption of brief interventions into routine practice? Implementation trials

Whilst there have been successive attempts to encourage the routine delivery of brief alcohol interventions in day-to-day practice, most efforts have demonstrated limited success (56–60), and implementation of this form of preventive care remains inconsistent. In the UK, for example, although survey data suggest that GPs see both preventative medicine and alcohol intervention as increasingly high priority public health areas, and they generally view primary health care as an appropriate setting to raise and discuss alcohol issues (61), most do not routinely ask patients about their drinking (62). In recognition of this mismatch, there has been an increased focus on implementation research to test potential approaches to improve their delivery (63).

Implementation studies may take a number of forms, exploring the many influences on patient, healthcare professional, and organizational behavior in either healthcare or population settings (63). In the alcohol intervention field, there has arguably been most progress in identifying the various obstacles experienced by practitioners seeking to deliver screening and brief alcohol interventions in routine primary health care. Some of the barriers to the provision of brief alcohol interventions identified to date concern the socio-cultural, interactional and attitudinal factors that influence their delivery by individual primary healthcare practitioners (64, 65). For example, there is an evidence to suggest that many GPs remain unconvinced that patients will heed advice to change their drinking behavior, particularly those patients drinking at heavier or dependent levels (66–68). Practitioners are also concerned that they might offend patients by discussing alcohol, or at least view alcohol as a “delicate” subject to raise in the standard consultation situation (65, 68), which potentially risks jeopardizing the patient–doctor relationship (69, 70). This “role insecurity” (71) may also relate to the potential impact that practitioners’ own drinking practices may have on intervention delivery, alongside confusion about what advice they should actually be delivering on lower risk drinking (61).

In addition, previous research also points toward a series of structural and organizational factors that influence alcohol intervention delivery. Lack of training or suitable intervention materials (68, 72), inadequate financial incentives (73, 74), unsupportive specialist alcohol service provision (3, 67), and everyday time pressures (67, 75) has all been identified by GPs and other health practitioners as barriers to their successful engagement in and delivery of brief interventions for alcohol (32, 59, 62, 64, 73, 76–79). Moreover, these barriers are often interrelated. Thus GPs’ discussions around alcohol are shaped by both the practical challenge of incorporating discussions about alcohol within the pressured, time-limited consultation process and their own (and the patient’s) complex social, cultural, and moral beliefs about what constitutes “normal” versus “problematic”drinking (64, 80, 81).

Alongside research to identify notable barriers to the routine delivery of screening and brief alcohol intervention in primary care, there have also been studies exploring facilitating factors. For example, Screening, Brief Intervention, and Referral to Treatment (SBIRT) is US-based program to promote the use of evidence-based practice to identify, reduce, and prevent problematic use, abuse, and dependence on alcohol and illicit drugs (82–86). One key message arising from this program of activities has been that effective training strategies for health professionals are an essential first step in the successful implementation of SBIRT, with team-based learning a potentially promising strategy to help maintain newly learned clinical skills (87). In addition, Ronzani et al. have shown the importance of involving managers in the dissemination of screening strategies and brief interventions to increase their implementation rates (88). Results from comparative work carried out in New Zealand, England, and Catalonia demonstrated the need to tailor procedures to fit with local circumstances, to break the process down into clinically acceptable steps, and to negotiate implementation strategies and timing taking into account local needs and competing demands to successfully embed intervention activity (89). The recent developments in the use of digitally mediated brief interventions (eBI) delivered via the internet and mobile phones represent another way of dealing with these issues (90). These offer practitioners a way to avoid the need to engage their patients directly in a discussion about alcohol, while at the same time providing an opportunity to reflect on their drinking behavior in a secure and confidential setting (see *Internet applications for screening and brief interventions for alcohol in primary care settings – implementation and sustainability* by Wallace and Bendtsen in this issue for more on this subject).

Regarding work that actively promotes uptake and adoption of brief interventions in practice, the largest study conducted to date was part of a World Health Organisation Collaborative project. This study found that active dissemination strategies were needed to ensure that practitioners were aware of the evidence on brief interventions, whilet both training and support were needed to convert this knowledge into action (60). Moreover, a systematic review and meta-analysis of strategies to engage practitioners in brief intervention activity found that a specific focus on alcohol *per se* and multi-component support programes were more effective (79) than focusing on several behavior and just a single education or support strategy. Bringing this field right up to date is the optimizing delivery of health care interventions (ODHIN) study, an ongoing Europe wide project involving research institutions from nine European countries. This trial has a factorial design and it aims to assess the impact on practitioner behavior of out-reach training, financial incentives, and the opportunity to refer patients to an electronic brief intervention programe, both individually and in differing combinations of approaches. This study is due to report in 2015.

#### Level 4: Wider roll-out work: Demonstration studies

A key limitation of earlier implementation research, however, is that changes in practitioner behavior tend to be limited to the time-frame of each individual study that attempts to promote screening and brief alcohol intervention. When the research work ends, the focus on screening and brief alcohol intervention also tends to stop. A significant challenge is to find ways of embedding this activity in mainstream clinical work to achieve sustained delivery (70), and also to be able to measure when and how often it occurs, and to whom it is delivered.

The development of national alcohol strategies, specific guidance for practitioners on when and how to deliver screening and brief interventions, and national payment programs for ASBI has recently been introduced in the UK to promote their wider roll-out (91, 92). Khadjesari et al. drew on routine UK general practice data (covering 382,609 patients, drawn from over 500 general practices) to examine the impact of financial incentives on the rates of screening for alcohol-use disorders (93). It found that following the introduction of screening incentives, relatively high rates of newly registered adult patients (76% nationally) were being screened for an alcohol-use disorder in English general practice settings. In addition, research conducted in the North East of England, which used routine data to compare recorded rates of delivery between general practices that were incentivized or non-incentivized for ASBI activity, determined that overall, practices associated with higher recorded rates of key ASBI service indicators were signed up to pay-for-performance schemes (94). Finally, and moving the field beyond the UK, the ongoing EU co-funded research brief interventions in the treatment of alcohol-use disorders in relevant settings (BISTAIRS) project seeks to intensify the implementation of brief alcohol intervention across Europe, including through the identification and dissemination of existing pockets of evidence-based good practice in established national primary health-care programs, with results from this work expected in 2015 (95).

## Conclusion

This paper demonstrates that overall, there is a plentiful outcome evaluation literature, which consistently reports positive effects of screening and brief alcohol intervention when delivered in primary care. Much of this literature is of a moderate to high quality, and the outcome effects persist even when the less well designed studies are discounted from the assessment. As such, we have surely long passed the point of needing to ask the question “do these interventions work?,” or even “do they work in the real world of primary care?.” Both efficacy and effectiveness have been comprehensively demonstrated through this substantial body of evidence, and intervention effects seem replicable and indeed stable over time, and across different study contexts. Indeed, with the benefit of hindsight, the brief intervention field seems to constitute an almost perfect example of the evaluation of a complex intervention (96). Early evaluations of screening and brief intervention approaches included more tightly controlled efficacy trials and were followed by more pragmatic trials of effectiveness in routine clinical practice. Attention then shifted to dissemination, implementation (60), and wider-scale roll-out (97). Nevertheless, we still seem to be a long way from consistent delivery of brief interventions to the majority of heavy drinking patients in routine primary care, and day-to-day implementation of this approach seems to be at best very modest (94). Moreover, while new studies appear at regular intervals in the published literature, these are still primarily focused on the assessment of intervention effects rather than on how to embed brief intervention approaches in mainstream care.

No field of research work is perfect however, and especially one that has been evolving over a 30 year period. Consequently, it is not surprising that a considerable degree of heterogeneity exists within the screening and brief intervention literature or that there can often seem to be a re-treading over previously covered ground. There is also a genuinely interesting and as yet unanswered question concerning what specific factors prompt the positive changes in alcohol consumption that occur after brief alcohol intervention that undoubtedly needs further examination. However, the search for these “active ingredients” should not delay progress in rolling out these interventions into health systems for patient benefit. Many people do not fully understand how their car actually works, yet most still successfully drive them each day. Given the positive evidence amassed to date, research efforts should maintain a continued focus on promoting sustained implementation of screening and brief alcohol intervention approaches in primary care.

Moreover, frontline practitioners responsible for the implementation of any policy or health program may make adaptations based on the availability of resources, compatibility with organizational or professional values, expertise, and knowledge (98), resulting in their “reinvention” of the intervention (99). The research community needs to accept this reality which might result in some loss of scientific purity (98). For the credibility of research in practice is judged less by its rigor than how it fits with professional wisdom and experience, and understanding of what “best evidence” actually means in day-to-day health care (100). Looking further forward, therefore, the key challenge for the brief intervention field in the future is to embrace translational research (101), in which academics, practitioners, and policy-makers work in closer partnership, potentially also with patients, in order to understand their world-view more clearly, and identify mutually acceptable ways of embedding brief interventions in practice (102).

## Conflict of Interest Statement

The authors declare that the research was conducted in the absence of any commercial or financial relationships that could be construed as a potential conflict of interest.
